# Increased ventilation by fish leads to a higher risk of parasitism

**DOI:** 10.1186/1756-3305-7-281

**Published:** 2014-06-23

**Authors:** Victor N Mikheev, Anna F Pasternak, E Tellervo Valtonen, Jouni Taskinen

**Affiliations:** 1 Laboratory of Behaviour of Lower Vertebrates, Institute of Ecology and Evolution, Russian Academy of Sciences, 33 Leninskii pr, 119071 Moscow, Russia; 2 Laboratory of Plankton Ecology, Institute of Oceanology Russian Academy of Sciences, 36 Nakhimovskii pr, 117997 Moscow, Russia; 3 Department of Biological and Environmental Science, University of Jyväskylä, PL 35, 40351 Jyväskylä, Finland

**Keywords:** *Diplostomum pseudospathaceum*, Rainbow trout, Ventilation rate, Oxygen concentration, Parasite acquisition

## Abstract

**Background:**

Fish are common intermediate hosts of trematode cercariae and their gills can potentially serve as important sites of penetration by these larval stages. We experimentally tested the hypothesis that volume of ventilation flow across the gills contributes to acquisition of these parasites by fish. We manipulated the intensity of ventilation by using different oxygen concentrations.

**Methods:**

Juvenile *Oncorhynchus mykiss* were individually exposed for 10 minutes to a standard dose of *Diplostomum pseudospathaceum* cercariae at three levels of oxygen concentration, 30, 60 and 90%. Ventilation amplitude (measured as a distance between left and right operculum), operculum beat rate, and the number of cercariae established in the eyes of fish were recorded.

**Results:**

Fish reacted to low oxygen concentration with wider expansion of opercula (but not with increasing beat rate), leading to an increase in ventilation volume. As expected, the intensity of infection increased with decreasing oxygen saturation—probably due to a higher exposure to cercariae caused by increased ventilation under low oxygen concentrations. The number of cercariae acquired by an individual fish was positively correlated with ventilation amplitude and with ventilation volume, but not with operculum beat rate. However, even though the infection rate increased under these circumstances, the proportion of larval trematodes successfully establishing in fish eyes decreased with increasing ventilation volume, suggesting that the high flow velocity, although increasing host exposure to cercarial parasites, may interfere with the ability of these parasites to penetrate their hosts. There was no difference in the behaviour of trematode cercariae exposed to low and high oxygen concentrations.

**Conclusion:**

A reduction in oxygen saturation resulted in an increase in ventilation volume across the gills and in doing so an increase in the exposure of fish to cercariae. A significant correlation between ventilation volume and parasitism represents the first experimental evidence that this physiological mechanism generates variation in transmission of parasites to fish hosts. Other factors that modify ventilation flow, e.g. physiological or social stressors, are expected to produce similar effects on the transmission success of the parasites penetrating fish hosts using the gills.

## Background

Respiration among most teleost fishes is based on pumping large volumes of water through the buccal and pharyngeal cavities and across the gills. This ventilation flow provides not only necessary dissolved oxygen, but contains various suspended particles including free living stages of parasites. Thus while gills serve as a principal organs for respiration, osmoregulation, and excretion [1], they also become a potentially important site of attachment and penetration by parasites. The importance of fish gills as penetration sites for *Diplostomum spathaceum* (Trematoda) cercariae was suggested by Whyte *et al*. [2] who observed considerably more metacercariae in the fish eye lenses when exposing the head part of the fish to cercariae rather than the rest of the body [2]. Studies of the migratory routes of diplostomules with radiometry and electron microscopy indeed showed that cercariae of *D. spathaceum* penetrate their fish hosts mainly through the gills. It was suggested that this “may be connected with fish ventilation hydrodynamics” [3]. Despite these strong indications that parasite penetration is associated with ventilation of fish, the role of ventilation rate in the infection process has received little attention. For example, there have been no experimental studies linking variations in parasite acquisition with changes in fish ventilation rates. Flow of the ventilation water in fish is regulated by two variables: ventilation frequency and the volume of each pump stroke [4]. Both parameters vary over a broad range [5]. Intensity of ventilation is highly variable and can be related to fish activity, hypoxia, and hypercapnia [4,5]. Various biotic and abiotic factors such as predator cues, feeding, pollution, and temperature can influence fish ventilation by either increasing or decreasing its rate [4,6-8]. Could intensity of infection by gill-penetrating parasites vary depending on ventilation rate and therefore be influenced by the internal and external factors affecting ventilation rate? How costly could increased ventilation be in terms of elevated parasite burden? To answer these questions, infection success of gill penetrating parasites should be examined while manipulating the ventilation activity.

Oxygen deficiency occurs extensively over large areas in freshwater ecosystems [9] and is also common in marine coastal systems [10]. Moreover, a global increase in hypoxia has been predicted to take place due to anthropogenic nutrient inputs and eutrophication [10]. In shallow waters with dense vegetation, oxygen deficiency is a common phenomenon during the summer, with oxygen levels dropping almost to zero during the night [11,12]. Oxygen deficiency is an important environmental stressor of aquatic organisms, including feral fish [13,14]. Water flow through the gill chamber changes considerably in response to the level of oxygen saturation [4,5]. With this in mind, we chose to use changes in water oxygen concentration as the means of manipulating ventilation rate.

Long-term effects of environmental stressors, including oxygen deficiency, on parasitism are well documented [15,16]. Increased parasite burden is primarily related to impaired immune responses that characteristically develop within several days or over longer periods [17-19]. Possible short-term effects (e.g., those taking place within hours) of acute oxygen deficiency on parasitism are unlikely to be based on immune response. A link between abrupt oxygen depletion and parasite acquisition would be expected if an increase in ventilation flow were to bring small passively floating trematode cercariae to the vicinity of fish gills.

The present study used rainbow trout, *Oncorhynchus mykiss*, to test whether a short-term reduction in oxygen concentration resulted in increased ventilation rate and thus increased parasite load when in the presence of cercariae of *Diplostomum pseudospathaceum.* We monitored operculum beat frequency and amplitude together with the number of acquired parasites at three levels of oxygen concentration. We also checked whether parasite activity was influenced by oxygen level. Our hypothesis was that ventilation by fish is an important variable linking parasitism and stress due to low oxygen levels.

## Methods

### Fish, parasites and infection procedure

*D. pseudospathaceum* is a common freshwater parasite that has a three-host life cycle, with snails and fish serving as intermediate hosts and piscivorous birds serving as definitive hosts [20,21]. *D. pseudospathaceum* is cosmopolitan in its distribution and infects at the stage of metacercariae in the eyes of many fish species including salmonids [22]. Rainbow trout, which is a commonly farmed fish in Finland, is known to serve as a suitable host for *D. pseudospathaceum*[23,24].

Experiments were carried out at Konnevesi Research Station, University of Jyväskylä. In the pilot experiment in August 2006, influence of oxygen depletion on infection with *Diplostomum* was observed. The main experiment designed to test whether ventilation by fish influences cercarial acquisition was carried out in August 2012. To ensure that fish used in this study had no parasites at the beginning of the experiments, rainbow trout aged 0+ year (fork length, mean ± SD: 8.6 ± 0.92 cm) were obtained from a commercial fish farm where they were reared in indoor tanks supplied with ground water. Fish were maintained in 1000 l flow-through tanks under a natural photoperiod, at 16-17°C, and fed with commercial food pellets (1.5 mm size, Nutra Parr LB, Norway). *D. pseudospathaceum* cercariae were obtained from 10 naturally infected *Lymnaea stagnalis* snails collected from Lake Konnevesi. *D. pseudospathaceum* is the only diplostomid species found in this snail in Lake Konnevesi [25,26]. The snails were allowed to produce cercariae for 2 to 4 hours. All cercariae were pooled into one suspension and the cercariae density was estimated by taking ten 1-ml samples. Cercariae were used for experiments within a period of 5 h from their emergence.

### Ventilation activity

Ventilation activity of fish was estimated while fish were maintained at three different concentrations of oxygen: 30% saturation (2.9 mg l^-1^), 60% saturation (5.5 mg l^-1^), and 90% saturation (9.2 mg l^-1^). To reduce oxygen levels, macrophytes from the lake were placed in four 40 l tanks for 4 hours. Two of the tanks were tightly covered and kept in the dark, while two additional tanks were illuminated and aerated. Within 5 hours, oxygen concentrations in the dark tanks decreased to 26-32%, while concentrations in the illuminated tanks ranged from 90 to 93%. We mixed water from the tanks with low and high oxygen concentrations to obtain water with intermediate levels of saturation (60%). Prior to our experiments water was filtered through 100 μm mesh to remove suspended debris.

Fish were individually placed into plastic boxes (24 × 16 × 10 cm) with two liters of water as soon as the concentration of oxygen was measured, and after 10 minutes were exposed to 400 cercariae at 17 – 17.5°C under illumination of 250 lux for another ten minutes. There was no difference in fish size, either in length (FL, mm) or in weight (g) between the different oxygen concentration treatments (One-way ANOVA: F_2,33_ = 0.397, *p* = 0.675 for FL and F_2,33_ = 0.529, *p* = 0.594 for weight). Following exposure to parasites, fish were taken out of the boxes and the final oxygen concentration was measured. Differences between the initial and final oxygen concentration did not exceed 1-3%. Fish were then kept individually in 8 l flow-through tanks for 2 days to allow *D. pseudospathaceum* metacercariae to develop to an easily recognizable size. Fish were killed by an overdose of MS222, measured, weighed, and inspected for the number of parasites in the eye lenses. A total of 36 fish were tested, 12 fish at each of the three levels of oxygen saturation.

To assess the ventilation activity of fish exposed to *D. pseudospathaceum* cercariae at the three levels of oxygen saturation, both the frequency and amplitude of opercula beats were recorded during the 10 min period of exposure to parasites by using video (Canon LEGRIA FS200) and photo (Nikon CoolPix 8.0) cameras that were mounted over the experimental boxes. Two minute video recordings and 15–20 photo frames were obtained for each fish. The number of opercula beats per minute was estimated from video recordings (opercula beat rate, OBR min^-1^). Three to five frames with the largest distance between the left and right opercula (amplitude) were chosen to estimate the “ventilation amplitude” (VA, mm). The means of these measurements were used for further analysis. The frequency of opercula beats was also recorded prior to cercariae introduction at the end of the 10 min acclimation.

Volume of a pump stroke (ml) was assessed for each fish using data on mean ventilation amplitude and approximating the volume of the water pumping chamber (ml) to a sphere with the diameter equal to ventilation amplitude. The volume of a pump stroke was calculated by subtracting the spherical volume of the chamber with closed opercula from the volume of the chamber when the opercula were open. To calculate ventilation volume (ml min^-1^), the volume of a pump stroke was multiplied by the number of opercula beats per minute.

The proportion of parasites established in the fish eyes was estimated by dividing the number of established parasites by the expected number of cercariae pumped with the water flow through the gills during the 10 min exposure. We assumed the distribution of cercariae within the water column was close to homogeneous. This is connected with the behaviour of both cercariae and fish. Cercariae interchange active hops to the surface with passive sinking to the bottom [27]. This leads to the dispersal of the parasites throughout the water column with slightly increased concentrations in the surface and bottom layers [28]. Fish movements and ventilation permanently mix the water in the experimental tanks causing more even distribution of cercariae. Given the negligible role of cercariae microdistribution, we expected that the number of parasites pumped through the buccal and pharyngeal cavities and across the gills would be directly proportional to the ventilation volume.

### Behaviour of cercariae

To check whether the activity of *D. pseudospathaceum* cercariae was influenced by water oxygen content, we examined the behaviour of individual parasites at 90 and 30% oxygen saturation. Several cercariae were transferred from a holding tank into a 5 ml chamber, where a randomly chosen parasite was observed for 2 min. A portion of these cercariae were then transferred to another chamber with another oxygen concentration. Time budget, the percentage of time when cercariae were immobile and activity level, the number of hops (bursts) were recorded. Ten cercariae at the high and low oxygen concentrations were examined.

### Data analysis

The data for infection rate and opercula beat rate and amplitude were tested with Shapiro-Wilk’s W test and met the assumptions of parametric testing. One-Way ANOVA (with Tukey HSD post-hoc tests) was used to analyze the effects of oxygen levels on parasite acquisition. Multiple regression and Spearman Rank Order correlations were used to examine relationships between infection intensity and ventilation activity. To compare burst rate and time budget of trematode cercariae under high and low oxygen saturation, a Mann–Whitney U test was used. In all cases, two-tailed tests were implemented.

### Ethical note

We used experimentally infected 0+ *O. mykiss*. The level of experimental infection in these studies was maintained at a much lower level than maximum values reported among natural populations of these fish hosts (up to 200–500 ind fish^-1^[29,30]). The level of mortality of infected fish in these experiments was low (less than 1%) and did not exceed that of control fish. No visible damages to fish were observed throughout the study. We minimized the required number of animals that were killed and dissected. Experimental fish were killed at the end of the tests with an overdose of MS 222, and dissected. The experiments were conducted with permission of the Lab-Animal Care and Use Committee of the University of Jyväskylä (licence number 30/30.5.2005 and ESAVI/6759/04.10.03/2011).

## Results

**Figure 1:**
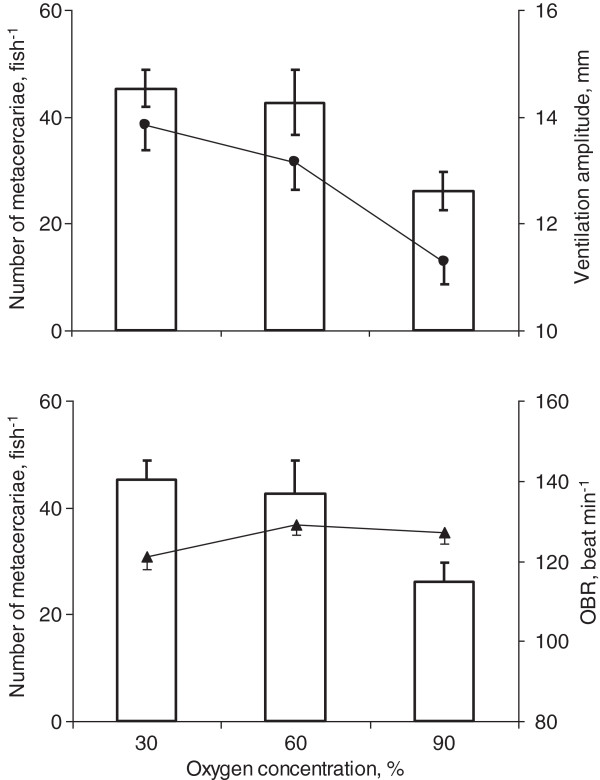
**Intensity of *Diplostomum pseudospathaceum *infection and characteristics of ventilation in *Oncorhynchus mykiss *at three levels of oxygen concentration. Upper panel**: number of metacercariae in the fish eyes (bars) and ventilation amplitude (dots) at 30, 60 and 90% oxygen concentration. **Lower panel**: number of metacercariae (bars) and operculum beat rate, OBR (dots) at 30, 60 and 90% oxygen concentration. Means and SE are shown.

Intensity of infection increased with a decrease in oxygen saturation (Figure 1). The effect of oxygen concentration on number of parasites was statistically significant (One-way ANOVA: F_2,33_ = 5.7, *p* = 0.007). The differences in intensity of infection between high (90%) and both medium (60%) and low (30%) oxygen saturation levels were significant (Tukey HSD test: *p* = 0.030 for medium *vs* high oxygen and *p* = 0.010 for low *vs* high oxygen). However, the difference between intensity of infection at medium and low oxygen concentrations was non-significant (Tukey HSD test: *p* = 0.902).

Fish reacted to low oxygen concentrations with a wider expansion of the opercula (Figure 1, upper panel). Ventilation amplitude significantly increased with lowering of oxygen saturation (One-way ANOVA: F_2,33_ = 5.7, *p* = 0.001). The differences in ventilation amplitude between high and both medium and low saturation levels were significant (Tukey HSD test: *p* = 0.019 for medium *vs* high oxygen and *p* = 0.001 for low *vs* high oxygen). No difference between ventilation amplitude at medium and low oxygen concentration levels was obtained (Tukey HSD test: *p* = 0.529). In addition, we found no difference in the operculum beat rate among fish when exposed to different concentrations of oxygen (Figure 1, lower panel) (One-way ANOVA: F_2,33_ = 2.2, *p* = 0.12), indicating that fish did not increase their operculum beat rate in response to decreased oxygen. No difference in operculum beat rate before and after introduction of parasites was found (Mann–Whitney U-test: *p* = 0.24 at 90%, 0.56 at 60 and 0.62 at 30% oxygen saturation). The data on fish exposed to parasites were used for later analysis.

**Figure 2:**
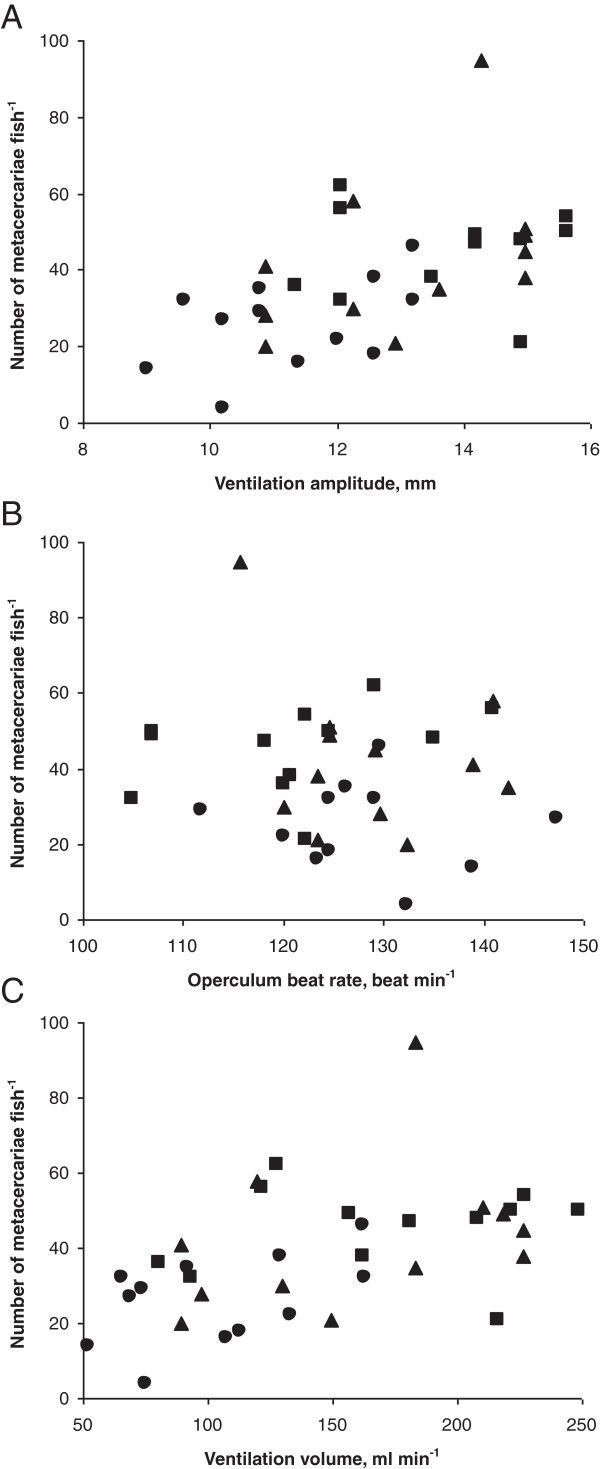
**Relationship between the number of acquired *Diplostomum pseudospathaceum *metacercariae and ventilation activity in *Oncorhynchus mykiss *at three levels of oxygen concentration. A**: number of metacercariae vs ventilation amplitude. **B**: number of metacercariae vs operculum beat rate. **C**: number of metacercariae vs ventilation volume. Squares – 30%, triangles – 60%, circles – 90% oxygen concentration.

The number of metacercariae acquired by individual fish was positively correlated with ventilation amplitude (Multiple regression: correlation = 0.52, p = 0.001) (Figure 2A). Correlations between infection intensity and ventilation amplitude were also positive within each O_2_ treatment condition, but statistically nonsignificant (Multiple regression: correlation = 0.43, 0.38, 0.11; p = 0.185, 0.252, 0.745 for 90, 60 and 30% oxygen concentration treatments, consequently). No correlation was found between operculum beat rate and parasite acquisition (Multiple regression: correlation = 0.06, p = 0.715) (Figure 2B). Operculum beat rate - infection intensity correlations were low and statistically insignificant within individual O_2_ treatments (Multiple regression: correlation = -0.04, -0.11, 0.36; p = 0.911, 0.756, 0.276 for 90, 60 and 30% oxygen concentration treatments, consequently).

Negative correlation between ventilation amplitude and operculum beat rate was not significant (Spearman Rank Order correlation: R_S_ = -0.30, p = 0.08).

Ventilation volume, calculated as the product of mean volume of pump strokes and mean rate of operculum beats, significantly increased with lowering of oxygen saturation (One-way ANOVA: F_2,33_ = 6.3, *p* = 0.005). The mean calculated volume of each pump stroke varied from 0.34 (high oxygen saturation) to 0.79 (medium oxygen saturation), and 0.99 ml fish^-1^ (low oxygen saturation), and represents 50, 103, and 128 ml kg^-1^ of fish mass, respectively. The differences between ventilation volume at high and both medium and low oxygen saturation levels were significant (Tukey HSD test: *p* = 0.022 for medium *vs* high oxygen and *p* = 0.006 for low *vs* high oxygen). No difference between ventilation volume at medium and low oxygen concentrations was obtained (Tukey HSD test: *p* = 0.870). The number of metacercariae acquired by individual fish was positively correlated with the ventilation volume (R_S_ = 0.56, p = 0.0006) (Figure 2C).

**Figure 3:**
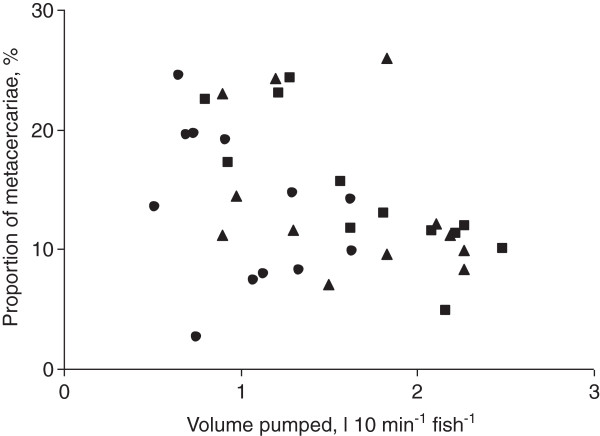
**Proportion of established *Diplostomum pseudospathaceum *metacercariae in relation to ventilation volume of *Oncorhynchus mykiss*.** Proportion was estimated as the percentage of established parasites of the number of cercariae pumped with the water flow through the gills during the period of exposure. Squares – 30%, triangles – 60%, circles – 90% oxygen concentration.

The higher ventilation volume correlated with a lower proportion of parasites established in the fish eyes (R_S_ = -0.39, p = 0.018) (Figure 3).

Behaviour of trematode cercariae was similar in both low and high oxygen treatments. Cercariae made short vigorous upward bursts, and then slowly sank down becoming immobile. No difference was found in either burst rates (they exhibited 4.25 ± 0.95 bursts min^-1^ at high oxygen concentration and – 4.55 ± 1.32 bursts min^-1^ at low oxygen concentration; Mann–Whitney U test: Z = 0.45, p = 0.650), or time budget (they stayed immobile for 78.8% and 76.8% of the time, respectively; Mann–Whitney U test: Z = -0.38, p = 0.705).

## Discussion

Reduction in oxygen saturation from 90 to 60% resulted in a considerable (1.63×) increase in parasite acquisition after only 10 min of exposure. A significant correlation between ventilation amplitude and the number of established parasites confirms that ventilation activity is an important variable affecting parasite acquisition and thus linking parasite acquisition with oxygen deficiency. This is the first experimental evidence to show that this physiological factor can generate variations in parasite transmission to fish hosts. Our results indicate directly that the number of established parasites depends on the volume of the water pumped through the gills. This supports the work of Höglund (1991) who suggested that increased concentrations of diplostomules in the gill area of fish may be connected with fish ventilation [3].

In contrast to many previous findings on the increased frequency of opercula beats in response to various environmental and physiological stressors [31-33], the results of our study found no significant changes in opercula beat rate when oxygen levels were reduced. Instead, we observed wider expansion of the opercula under low oxygen, which resulted in an increase in the volume of each pump stroke. This way of increasing the ventilation volume is considered to be of high importance [4,34,35]. Negative correlation between opercula beat rate and width of the expansion of the opercula suggests that fish are able to use either change of beat rate or expansion of opercula to control ventilation.

Either way, cercariae would presumably benefit from elevated water flow through the gills as they are poor swimmers and must be brought passively to the gills in order to penetrate these tissues. Their intermittent swimming consists of upward-directed active swimming and a passive sinking phase [36,37]. *Diplostomum* cercariae come into contact with the fish hosts by chance while maintaining their position in the water column [3,38]. The transmission success of cercariae depends mainly on accidental contact with the host. In general, trematode cercariae can actively move toward their host’s surface only over a few millimeters [39]. Fish ventilation activity producing directional water flow towards the gills provides parasites with a powerful mechanism of transportation in a viscous media. Interestingly, if parasites drifting in the ventilation flow miss the gills, their main entrance into the host [2,3], they are still in the vicinity of the surface of the skin and may penetrate through this tissue. Thus, all factors affecting ventilation, including various physiological factors and social stressors, could be considered as important modifiers of transmission of cercariae to their hosts.

Oxygen deficiency is common in aquatic ecosystems [9,10] and is known to cause increased ventilation in fish [5,35]. The present results show that most rapid increase in ventilation and subsequent acquisition of *D. pseudospathaceum* cercariae occurred with a moderate reduction of oxygen concentration. Further decrease in oxygen did not cause a significant effect. This can be related either to a progressively reduced increase in ventilation with decreased oxygen concentration or to a decrease in the proportion of cercariae penetrating fish tissues at higher water flow velocity. Our results support both hypotheses. Ventilation amplitude, the major determinant of the ventilation volume in the current experiments, increased more when oxygen was reduced from 90 to 60% than when it was reduced from 60 to 30% (Figure 1). Furthermore, the proportion of the established metacercariae, estimated as the percent of those pumped through the gills, decreased with an increase in ventilation volume (Figure 3). Thus, even though the infection rate increased, the proportion of successfully established parasites decreased with increasing ventilation volume, suggesting that high flow velocity, although increasing host exposure to cercariae, interferes with a cercaria’s ability to penetrate a host. According to this scenario, an increase from low to moderate ventilation flow would increase the exposure to parasitism by increasing the contact rate between cercariae and host fish, but at very high ventilation flow the high contact rate would be counterbalanced by a decrease in the penetration abilities of cercariae.

Other environmental stressors (e.g., pollution and suboptimal temperature) can also influence ventilation rates [4,31,40,41] and thus could modify the transmission of parasites to fish. Ventilation rate is a convenient and sensitive tool for assessing both alertness and stress levels in fish. Ventilation rate is also rapidly altered in response to social stress [42], handling and confinement [7,8], and under predation risk [43,44]. Stress due to human impact may thus increase exposure of fish to parasitism both in natural populations and under fish farming conditions.

Transmission success and efficiency of host manipulation by parasites depends on the number of penetrated parasites [45,46]. In the fish – *Diplostomum* system, the more cercariae that accumulated in a fish, then the more deleterious are the expected effects [24,47] as is the manipulation of the fish [48]. Thus, variability in the ventilation rate, connected to personality traits [49], social interactions [42], or irritation by previously acquired parasites [40], can be among the causes of variation in the parasite loads of fish. For example, it has recently been shown that fish in shoals exhibit a lower risk of infection by trematode cercariae than solitary fish, due to a more efficient avoidance of parasitized habitats in shoals [28] or because they seek shelter against parasite larvae in the centre of shoal [50]. Furthermore, under experimental conditions a solitary fish can be more stressed than fish in a group. Indeed, Stumbo *et al.*[50] stated that the role of experimental factors that differentially affect parasitism among solitary vs. grouped fish cannot be ruled out “until we know more about the linkage between stress and recruitment of metacercariae in fish” [50]. We think that our results provide such a link, a direct connection between physiological stress and exposure to parasites, mediated by changes in ventilation rates by fish.

Low oxygen stress may lead to the increased susceptibility of fish to parasites. The duration of the impact is of primary importance. While long-term (days to weeks) effects of oxygen deficiency on parasitism have been shown [15,16], the short-term effects (minutes to hours) are much less studied. Usually, increased infection rate is explained through impaired immune responses that develop within several days or over longer periods [17-19]. That is why short-term effects that lead to increased parasite burden are hardly based on impaired immunity. Therefore, the present short-term variations in acquisition of cercariae are probably related to the stress-induced changes of ventilation rate.

There are many trematode species with tiny free-swimming stages, which have to find and penetrate their fish host. Like the cercariae of *Diplostomum*, they could also benefit from the ventilation flow that brings them closer to their host. The significance of this ventilation-based mechanism of parasite transportation may be particularly high in stagnant habitats where molluscs, the first intermediate hosts of many trematodes, are abundant. When studying host-parasite interactions between trematodes and fish, ventilation rates and factors affecting these rates should be considered as important determinants of parasite transmission.

## Conclusion

Our results strongly suggest that ventilation flow across fish gills affects transmission success of *D. pseudospathaceum* cercariae to the second intermediate hosts. Ventilation flow that brings free-swimming cercariae to the fish gills, the main site of penetration, is highly variable depending on changes in oxygen concentration, an important environmental stressor. In our experiments, the number of metacercariae acquired by juvenile rainbow trout markedly increased with a decrease in oxygen concentration. Long-term effects of oxygen depletion that are known to increase the risk of parasitism are related to the impairment of the host immune system. We found that a considerable increase of infection intensity can be caused by a short-term increase in ventilation flow induced by oxygen deficiency. This simple physiological mechanism could be a powerful factor influencing the variability in acquisition of free-swimming parasites in aquatic systems like heterogeneous shallow water areas and fish farms where pronounced changes of oxygen often occur. In addition, other stressors that modify ventilation flow are expected to produce similar effects on the transmission success of the parasites penetrating through the gills. Thus, various biotic (e.g., predation threat, social interactions) and abiotic (e.g., oxygen, temperature) stressors, which are important modifiers of ventilation flow are suggested to play a significant role in parasite acquisition by fish.

## Competing interests

The authors declare that they have no competing interests.

## Authors’ contributions

VM and AP conceived the study, performed the experiments, and wrote the manuscript. JT contributed to planning and implementation of the study, and clarified the manuscript. ETV conceived and supervised the study. All authors read and approved the final manuscript.
